# The RIO protein kinase-encoding gene *Sj-riok-2* is involved in key reproductive processes in *Schistosoma japonicum*

**DOI:** 10.1186/s13071-017-2524-7

**Published:** 2017-12-12

**Authors:** Lu Zhao, Xin He, Christoph G. Grevelding, Qing Ye, Ying Li, Robin B. Gasser, Colette Dissous, Mudassar N. Mughal, Yan-Qin Zhou, Jun-Long Zhao, Min Hu

**Affiliations:** 1 State Key Laboratory of Agricultural Microbiology, College of Veterinary Medicine, Huazhong Agricultural University, Wuhan, People’s Republic of China; 2 Institute of Parasitology, BFS, Justus-Liebig-University Giessen, Giessen, Germany; 3 Faculty of Veterinary and Agricultural Sciences, The University of Melbourne, Parkville, Melbourne, Australia; 4 CIIL - Center for Infection and Immunity of Lille Inserm, University Lille, Lille, France

**Keywords:** *Schistosoma japonicum*, RIO2 kinase, RNA interference, Gonad, Reproductive development

## Abstract

**Background:**

Schistosomiasis is one of the most prevalent parasitic diseases worldwide and is caused by parasitic trematodes of the genus *Schistosoma*. The pathogenesis of schistosomiasis is caused by eggs whose production is the consequence of the pairing of schistosomes and the subsequent sexual maturation of the female. Previous studies have demonstrated that protein kinases are involved in processes leading to the male-induced differentiation of the female gonads, ovary and vitellarium**.** Right open reading frame protein kinase 2 (RIOK-2) is a member of the atypical kinase family and shown in other organisms to be responsible for ribosomal RNA biogenesis and cell-cycle progression, as well as involves in nematode development. However, nothing is known about its functions in any trematode including schistosome.

**Methods:**

We isolated and characterized the *riok-2* gene from *S. japonicum*, and detected the transcriptional profiles of *Sj-riok-2* by using real-time PCR and *in situ* hybridization. RNAi-mediated knockdown of *Sj-riok-2* was performed, mitotic activities were detected by EdU incorporation assay and morphological changes on organs were observed by confocal laser scanning microscope (CLSM).

**Results:**

*In silico* analyses of the amino acid sequence of *Sj*-RIOK-2 revealed typical features of this class of kinases including a winged helix (wHTH) domain and a RIO kinase domain. *Sj-riok-2* is transcribed in different developmental stages of *S. japonicum*, with a higher abundance in adult females and eggs. Localization studies showed that *Sj-riok-2* was mainly transcribed in female reproductive organs. Experiments with adult schistosomes in vitro demonstrated that the transcriptional level of *Sj-riok-2* was affected by pairing. Knocking down *Sj-riok-2* by RNAi reduced cell proliferation in the vitellarium and caused the increased amount of mature oocytes in ovary and an accumulation of eggs within the uterus.

**Conclusions:**

*Sj-riok-2* is involved in the reproductive development and maturation of female *S. japonicum*. Our findings provide first evidence for a pairing-dependent role of *Sj-riok-2* in the reproductive development and maturation of female *S. japonicum.* Thus this study contributes to the understanding of molecular processes controlling reproduction in schistosomes.

**Electronic supplementary material:**

The online version of this article (10.1186/s13071-017-2524-7) contains supplementary material, which is available to authorized users.

## Background

Schistosomiasis is a serious disease caused by parasitic flatworms of the genus *Schistosoma*, and it affects more than 230 million people in 78 countries around the world [1]. The control of this disease mainly relies on chemotherapy, as no vaccine is available [2]. However, although the drug praziquantel (PZQ) is widely used against all *Schistosoma* species, it cannot prevent re-infection [3, 4]. In addition, accumulated evidence indicates that resistance to this drug might occur [5–10]. Therefore, alternative treatment strategies are required for controlling the parasite. To this end, research activities aim at understanding biological processes in schistosomes at the molecular level. As schistosomiasis is associated with granulomatous inflammatory responses in different host tissues, including liver, the intestinal tract and spleen (hepato-intestinal form) or the urinary bladder and/or genital tract (urogenital form), are induced by eggs deposited by female schistosomes [11], research efforts have focused on identifying processes responsible for growth and reproduction. The latter is of specific interest, because the pairing of females with male schistosomes is unique with respect to physiological consequences. Unpaired, virgin-like females possess stem cell-like precursor vitelline cells and a small ovary containing stem cell-like precursor oocytes, the oogonia. Pairing induces mitoses and differentiation processes that lead to the completion of the development of the female gonads, ovary and vitellarium. Both organs deliver cells, mature oocytes and S4 vitelline cells, respectively, needed for the synthesis of composite eggs [12–14]. Among others, signal transduction processes have been detected to be involved in male-female interactions controlling oocyte and vitelline cell differentiation [15–17].

In this context, cellular protein kinases PKs represent intensively studied class of molecules. As demonstrated in model organisms and eukaryotic cell cultures, PKs are essential for regulating various cellular processes, such as transcription, cell cycle progression, cytoskeletal rearrangement and metabolic processes [18, 19]. Based on their structure, PKs can be classified into eukaryotic protein kinase (ePK) and atypical protein kinase (aPK) [18, 19]. More than 90% of human PKs are ePKs, which are characterized by the presence of a “catalytic domain”, containing 11 major conserved sub-domains which are separated by regions of lower conservation and important for substrate binding [18]. The aPKs share a homologous catalytic core-structure with ePKs, but exhibit limited overall sequence similarity [20]. In schistosomes, several ePKs such as Src-like tyrosine PKs, Syk-like PK, Src/Fyn-like hybrid PK, Src/Abl- and Abl-like PKs, cyclic guanosine-3′,5′-monophosphate dependent protein kinase (cGKs), mitogen-activated PKs (MAP), protein kinases C (PKCs), extracellular signal-regulated kinases (ERKs) and Polo-like kinase (PLK) were found to be specifically or predominantly expressed in gonads, and proven to be involved in reproductive development and egg production by functional analyses [21–31]. Although the Venus kinase receptor (an atypical receptor tyrosine kinase) and an atypical protein kinase C had been demonstrated to play important roles in the reproduction and viability of schistosomes [32], there is still limited information about the functions or roles of other aPKs in reproductive development in schistosomes.

One family of aPKs are the RIO (right open reading frame) kinases, which were first characterized in yeast [33]. To date, four members of the RIO-kinase family, RIOK-1, RIOK-2, RIOK-3 and RIOK-B, have been identified [34, 35]. As a non-ribosomal factor required for normal ribosomal RNA biogenesis, RIOK-2 has been shown to be indispensable for 18S pre-rRNA production and cytoplasmic 40S ribosomal subunit maturation in both yeast and human cells [36–39]. In yeast, RIOK-2 as an assembly factor is associated with pre-40S subunit to prevent premature translation initiation [40, 41]. In addition, RIOK-2 exerts important roles in cell cycle progression in different cancer cells [42, 43]. The RIOK-2-encoding gene *riok-2* is transcribed in different developmental stages of parasitic nematodes [44, 45] and is crucial for larval growth and development in *Caenorhabditis elegans* [46, 47]. However, nothing is known about the function(s) of RIOK-2 in any trematode.

In the present study, we characterized the RIOK-2-encoding gene of *Schistosoma japonicum* (*Sj-riok-2*). In addition to cloning, *in silico* analyses, and transcriptional profiling, different physiological and morphological techniques were applied to demonstrate that *Sj-riok-2* contributes to male-female interaction in *S. japonicum* and to important roles in female reproductive development and fertility.

## Methods

### Parasite material

Snails (*Oncomelania hupensis*) infected with *S. japonicum* (Jiangsu Provincial Institute of Parasitic Disease Control and Prevention, China) were maintained in water and illuminated for 2–4 h at 25 °C to induce the shedding of cercariae [48]. Six- to eight-week-old Kunming female mice (Hubei Provincial Center for Disease Control, China) were infected percutaneously with 2000–3000 cercariae (for the subsequent collection of skin- and lung-stage schistosomula) or 30 ± 5 cercariae (for the collection of hepato-portal schistosomula, adults and eggs) per mouse. Skin-stage schistosomula were recovered directly from crushed skin from the mice infected for 3 h and incubated with Earle’s equilibrium liquid. Lung-stage schistosomula were collected from crushed lung pieces incubated with Earle’s equilibrium liquid following right ventricular-pulmonary artery perfusion of the mice that had been infected for 3 d [48]. Hepato-portal schistosomula and adults were collected by hepato-portal perfusion of mice that had been infected for 21, 28, 35, 42 and 49 d, respectively [48]. Eggs were harvested from the livers of the mice infected for 35–49 d by comminution and layered-filtration with nylon mesh [48, 49]. For total RNA isolation, individual developmental stages collected were washed with diethypyrocarbonate (DEPC) water twice and preserved in TRIzol Reagent (Invitrogen, Carlsbad, CA) at −80 °C.

### *In silico* analyses

The expressed sequence tag (EST) sequence of *Sj-riok-2* (GenBank accession no. AY814262.1) was obtained by using the BlastN algorithm (https://blast.ncbi.nlm.nih.gov/Blast.cgi). The cDNA sequence that mapped to the gene in the draft genome was obtained from the *S. japonicum* genome of WormBase ParaSite (http://parasite.wormbase.org/ftp.html). Exon-intron boundaries in genomic sequences were determined by blast analyses using cDNA sequences as probes. The *Sj*-RIOK-2 protein sequence was aligned with orthologs of selected species by employing PROSITE [50] (www.expasy.ch/tools/scnpsit1.html), Pfam [51] (www.sanger.ac.uk/Software/Pfam/), and Clustal Omega alignment of European Bioinformatics Institute (www.ebi.ac.uk/Tools/msa/clustalo/). Functional domains and sub-domains were identified based on the protein alignment.

The amino acid sequences of 14 orthologs were retrieved from the GenBank database for further phylogenetic analysis, which was performed by using the MEGA 5 neighbor joining (NJ) method [52]. The species selected were nine invertebrates including *Brugia malayi* (CRZ23003.1), *Clonorchis sinensis* (GAA29846.2), *Caenorhabditis elegans* (CAC70109.2), *Drosophila melanogaster* (NP_651365.1), *Haemonchus contorts* (ADW27445.1), *Loa loa* (EFO24525.1), *Schistosoma haematobium* (XP_012792720.1), *S. mansoni* (CCD79377.1), *Strongyloides ratti* (CEF64700.1) and four vertebrates including *Danio rerio* (NP_998719.2), *Homo sapiens* (NP_060813.2), *Mus musculus* (NP_080210.1), *Xenopus tropicalis* (NP_001016682.1), and yeast *Saccharomyces cerevisiae* (KZV08416.1). Confidence limits were assessed by bootstrapping using 1000 pseudo-replicates for NJ, and other settings were obtained using the default values in MEGA v.5.0. A 50% cut-off value was implemented for the consensus tree [52].

### Rapid amplification of cDNA ends (RACE) and cloning of the *Sj-riok-2* cDNA

Total RNA of adult *S. japonicum* worms was isolated by TRIzol (Invitrogen, NY, USA) according to the manufacturer’s instructions. Total 3′-ends cDNA was synthesized using *Sj-riok-2*-specific internal primers (Sjriok2-1F and Sjriok2-3F, Additional file 1: Table S1) using the SMARTer™ RACE cDNA Amplification Kit (Clontech Laboratories, CA, USA). The cycling conditions for 3′ RACE-PCR were as follows: denaturation at 94 °C for 3 min followed by 5 cycles at 66 °C/63 °C for 30 s, 72 °C for 2 min, and 30 cycles at 94 °C for 30 s, 64 °C/61 °C for 30 s, 72 °C for 2 min, with a final extension at 72 °C for 10 min. After cloning and sequencing, the sequence amplified by 3′ RACE-PCR was merged to the known sequence region to generate a full-length *Sj-riok-2* sequence. For verification of the entire putative open reading frame (ORF), two additional primers Sjriok2-ORF-F and Sjriok2-ORF-R (Additional file 1: Table S1) were designed based on the reconstructed full-length sequence. The amplification conditions for the PCR following the reverse-transcription reaction (PrimeScript 1st Strand cDNA Synthesis Kit) were as follows: 94 °C for 3 min followed by 35 cycles at 94 °C for 30 s, 60 °C for 30 s, 72 °C for 2 min, with a final extension at 72 °C for 10 min. The PCR product was cloned into pMD19-T (Takara Biotechnology, Dalian, China) and then sequenced to confirm the integrity of the full-length sequence. The full *Sj-riok-2* cDNA sequence was deposited in GenBank with the accession no. KY884990.

### Quantitative real time PCR

Total RNA was isolated from cercariae, skin and lung-stage schistosomula, hepato-portal worms, adult female and adult male worms as well as eggs. The cDNAs were produced by RNA reverse transcription following PrimeScript™ RT Reagent Kit with the gDNA Eraser procedure (Takara, Dalian, China) and used as templates for PCR amplification using the SYBR Green PCR Master Mix (Takara, Dalian, China) and the ABI 7500 detection system (Applied Biosystems, USA). β-tubulin was used as an internal standard control (GenBank: AY220457.2) [53, 54]. Two pairs of primers were designed to *Sj-riok-2* (riok2-F and riok2-R), *Sj-plk-1* (plk1-F and plk1-R) and *Sj-β-tubulin* (β-Tubulin-F and β-Tubulin-R), respectively (Additional file 1: Table S1). Reactions (10 μl) were repeated in triplicate under the following conditions: 50 °C for 2 min, followed by 95 °C for 30 s, then 40 cycles of 95 °C for 15 s, 58 °C for 15 s, and 72 °C for 20 s, followed by a final 10 min elongation step at 72 °C. For the analysis of the data and graphical representation, the 2^-△△Ct^ method was used [55]. Cercariae were used as a calibrator and statistical analyses were given as the mean ± standard deviation and differences among different stages by using One-Way Analysis of Variance (ANOVA) for statistical analysis. A *P* value of ≤ 0.05 was considered as statistically significant.

### *In situ* hybridization

*In situ* hybridization experiments were performed to localize *Sj-riok-2* mRNA in male and female parasites using an established approach [26, 56]. In brief, adult worm pairs were fixed in Bouin’s solution (picric acid/ acetic acid/formaldehyde; 15/1/5) and subsequently embedded in paraplast (Sinopharm) overnight. Five micrometers paraffin-sections of adult worms were incubated in xylol before rehydration with a graded ethanol series (100–30%) and then treated with proteinase K to remove protein (final concentration 1 μg/ml). For RNA probe preparation, complementary DNA fragments of *Sj-riok-2* (nt positions 1146–1481) and an eggshell gene (p14 ortholog from *S. mansoni*) of *S. japonicum* (*Sj-*es, GenBank: M59318.1; nt positions 440–809) [57] were synthesized to produce templates for probe labeling by PCR with the primers tagged with T7 and SP6 promoter sequences (underlined bases) (Additional file 1: Table S1). Sense and antisense transcripts were labeled with digoxigenin following the instructions of the manufacturer (Roche Applied Science, Basel, Switzerland). Labeled transcripts of *Sj-riok-2* and *Sj-es* gene were size-controlled by gel electrophoresis. The sections were hybridized in hybridization solution containing 100 μl of deionized formamide, 50 μl of 20× SSC, 4 μl of 50× Denhardt’s solution, 2 μl of Tween 20, 4 μl of denatured salmon sperm DNA (10 mg/ml), 8 μl of yeast RNA (33 mg/ml) and 6 μl of each specific antisense or sense probe. The probes were heated at 70 °C for 10 min before use and DEPC-treated H_2_O added to a final volume of 200 μl at 42 °C for 16 h in a wet chamber containing 20% glycerol. Then, the sections were washed with 2×SSC, 1×SSC and 0.5×SSC for 15 min each and blocked with 2% blocking reagent (Roche Applied Science, Basel, Switzerland) at 22–25 °C (room temperature, RT) for 30 min. The detection of digoxigenin-labeled transcript was done using alkaline phosphatase conjugated anti-digoxigenin antibodies (1:1000) in 1% blocking solution. Unbound antibodies were removed by washing with maleic acid solution twice for 20 min each. Then colour reaction was developed with naphthol-AS-phosphate and Fast Red TR (Sigma, USA) in the dark. The worm sections were covered with a cover slip and sealed with water soluble sealing medium (Boster, China) and air-dried. Finally, the staining location and intensity were observed under microscope (E80i, Nikon, Japan) at 20- and 40-times magnification.

### Pairing experiments in vitro

Adult worms collected from mice 42 d after infection were cultured at 37 °C in vitro in 841 medium (pH 7.4) consisting of RPMI 1640 medium (Invitrogen, USA) supplemented with 10% *V/V* new born calf serum (Gibco, New Zealand), 1 mg/ml lactalbumin hydrolysate, 10^−6^ mol/l hydrocortisone, 5 × 10^−7^ mol/l hypoxanthine, 10^−6^ mol/l serotonin, 100 U/ml penicillin and 100 μg/ml streptomycin.

For pairing experiments, 10 pairs of worms as well as 10 single females and 10 single males were cultured in supplemented 841 medium for 3 d and 9 d. The medium was changed every 2 days. In addition to paired cultured and single cultured groups, the re-mating group was also set up. At first, single females and single males were cultured separately for 2 d. On the third day, single females and males were co-cultured together for 24 h, and re-mated pairs were selected for continued culturing for 6 d. At each time point (3 d and 9 d), couples (for control), re-paired worms, single females or single males were collected for real-time PCR analyses. Female and male worms from couples were used as calibrators, and statistically significant difference were determined by two-way ANOVA analysis.

### RNAi-mediated knock down of *Sj-riok-2* expression in vitro

Complementary DNA fragments of *Sj-riok-2* (1811 bp) and *Sj-plk-1* (1701 bp, PLK-1-encoding gene of *S. japonicum*) were synthesized, respectively, to produce templates (for dsRNA synthesis) by PCR with the following primer combinations: dsRNA-riok2-F/dsRNA-riok2-R and dsRNA-plk1-F/ dsRNA-plk1-R tagged with T7 and SP6 promoter sequences (underlined bases) (Additional file 1: Table S1).The primer pair dsRNA-stk6-F and dsRNA-stk6-R (Additional file 1: Table S1) designed to amplify *Sj-stk-6* (Tyrosine kinase 6 encoding gene of *S. japonicum*) generated a 1111 bp product that was used as a negative-control. The cycling conditions for the amplification of the *Sj-riok-2* region were an initial denaturation step at 94 °C for 3 min, followed by 35 cycles at 94 °C for 40 s, 64 °C for 40 s and 72 °C for 2 min, with a final extension at 72 °C for 10 min. The cycling conditions for *Sj-plk-1* were an initial denaturation step at 94 °C for 3 min, followed by 5 cycles at 94 °C for 40 s, 61 °C for 30 s and 72 °C for 2 min, 30 cycles at 94 °C for 30 s, 63 °C for 30 s and 72 °C for 2 min with a final extension at 72 °C for 10 min. The cycling conditions for *Sj-stk-6* were an initial denaturation step at 94 °C for 3 min followed by 35 cycles at 94 °C for 40 s, 65 °C for 40 s and 72 °C for 2 min, with a final extension at 72 °C for 10 min. Each PCR product was cloned into pMD-19 T and directly sequenced. Linearized plasmids obtained by a restriction enzyme downstream of the vector were used as templates for in vitro transcriptions to generate single-stranded RNAs. The reaction volume (20 μl) contained 1.5 μg of linearized plasmids, 2 μl 10× Reaction Buffer, 2 μl A/C/G/UTP T7 and 2 μl T7/SP6 RNA polymerase mix (MEGAscript RNA transcription kit, Invitrogen, USA). Equal amounts of the single-stranded RNAs were mixed into annealing buffer (500 mM potassium acetate, 19 mM magnesium acetate, 150 mM HEPES-KOH, pH 7.4) and incubated at 90 °C for 8 min and subsequently placed at RT for 2–4 h. The integrities of ssRNA and dsRNA molecules were confirmed by agarose gel electrophoresis.

For RNAi experiments, 28 d old schistosomes were cultured in 6-well cell-culture plates, and each well contained five worm couples in 5 ml medium 841 and 10–20 μg dsRNA. The experimental setup comprised different groups of worms including couples treated with 10 μg *Sj-riok-2* dsRNA, or 10 μg *Sj-plk-1* dsRNA, or 10 μg *Sj-riok-2* plus 10 μg *Sj-plk-1* dsRNA, respectively. Control groups included couples treated with 10 μg *Sj-stk-6* dsRNA, which is directed against a non-related Src/Abl hybrid kinase [22] or couples which were not treated with any dsRNA (control). All parasites were cultured at 37 °C for 9 d. The culture medium was changed every 2 days (the new culture media and dsRNA were added together to replace the old media). Parasites cultured for 9 d were harvested for real-time PCR analysis of *Sj-riok-2* mRNA level and morphological analysis. Non-treated females and males were used as calibrators, and statistically significant differences were determined by two-way ANOVA analysis.

### 5-ethynyl-2′-deoxyuridine (EdU)-incorporation assay

Paired worms treated with dsRNA for 9 d were incubated with 10 μM of the thymidine analog EdU in medium 841 for 24 h. EdU incorporation was detected by using Click-iT® EdU Imaging Kits (Invitrogen, USA) using a previously described method [58]. Paired worms were separated and fixed in phosphate-buffered saline (PBS) containing 0.3% Triton X-100 (PBSTx) and 4% paraformaldehyde for 6 h at RT. After being rinsed in PBSTx, the worms were dehydrated in 50% and 100% methanol for 10 min. Then, worms were rehydrated with 50% methanol and washed in PBSTx for 10 min. Protease K (6 μg/ml) was added to the worms, which were kept for 25 min at RT for protein digestion. Subsequently, the worms were fixed with 4% paraformaldehyde in PBSTx for 10 min and then rinsed twice in PBS containing 3% BSA. After being incubated in the click-it reaction cocktail for 30 min (shaking), the worms were rinsed twice again in PBS containing 3% BSA and stained with Hoechst 33342 (diluted 1:2000 in PBS) in the dark for 30 min at RT or overnight at 4 °C. The specimens were examined by confocal laser scanning microcopy (CLSM) using an Olympus FV1000 confocal microscope with 405 nm (for Hoechst) and 633 nm (for Alexafluor 647) laser stimulation, respectively, after being washed and mounted with Anti-fade Mounting Medium (Beyotime, China).

### Morphological analysis

The procedure was performed as previously described [21] with minor modifications. Briefly, collected worms were fixed with AFA (95% ethanol, 3% formaldehyde and 2% glacial acetic acid) for at least 24 h and stained with 0.25% chloride carmine (Solarbio, China) for 30 min. After destaining in 70% ethanol with 1% hydrochloric acid, the worms were dehydrated in 70%, 90% and 100% ethanol for 3 min each. Then, worms were cleared in methyl salicylate and preserved as whole mounts with neutral resins on glass slides. Specimens were examined using a confocal laser microscope (Olympus FV1000) under reflection mode employing a 488 nm He/Ne laser. 

## Results

### Cloning and sequence analysis suggest that *Sj-riok-2* is a member of the RIO-kinase family

**Fig. 1:**
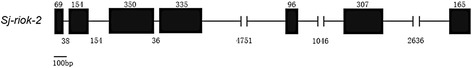
Schematic diagram showing the genomic organization of *riok-2* of *Schistosoma japonicum*. Black boxes indicate exons, with the numbers above indicating the length (bp) of an exon. Introns are indicated by slanted lines between the exons, with the numbers indicating intron length (bp)

The cDNA of *Sj-riok-2* (GenBank: KY884990) is 2164 bp long, containing a 1476 bp open reading frame (ORF) that encodes 491 amino acids, a 344 bp 5′-UTR, and a 344 bp 3′-UTR including the poly-A tail preceded by a non-canonical poly-adenylation signal (ATTAAA) 40 bp upstream. The genomic DNA of *Sj-riok-2* is more than 11,082 bp in length, and is composed of 7 exons varying from 69 bp to 350 bp in length and 6 introns varying from 36 bp to 4751 bp in length (Fig. 1). All exon-intron junctions follow the GT-AG rule.

**Fig. 2:**
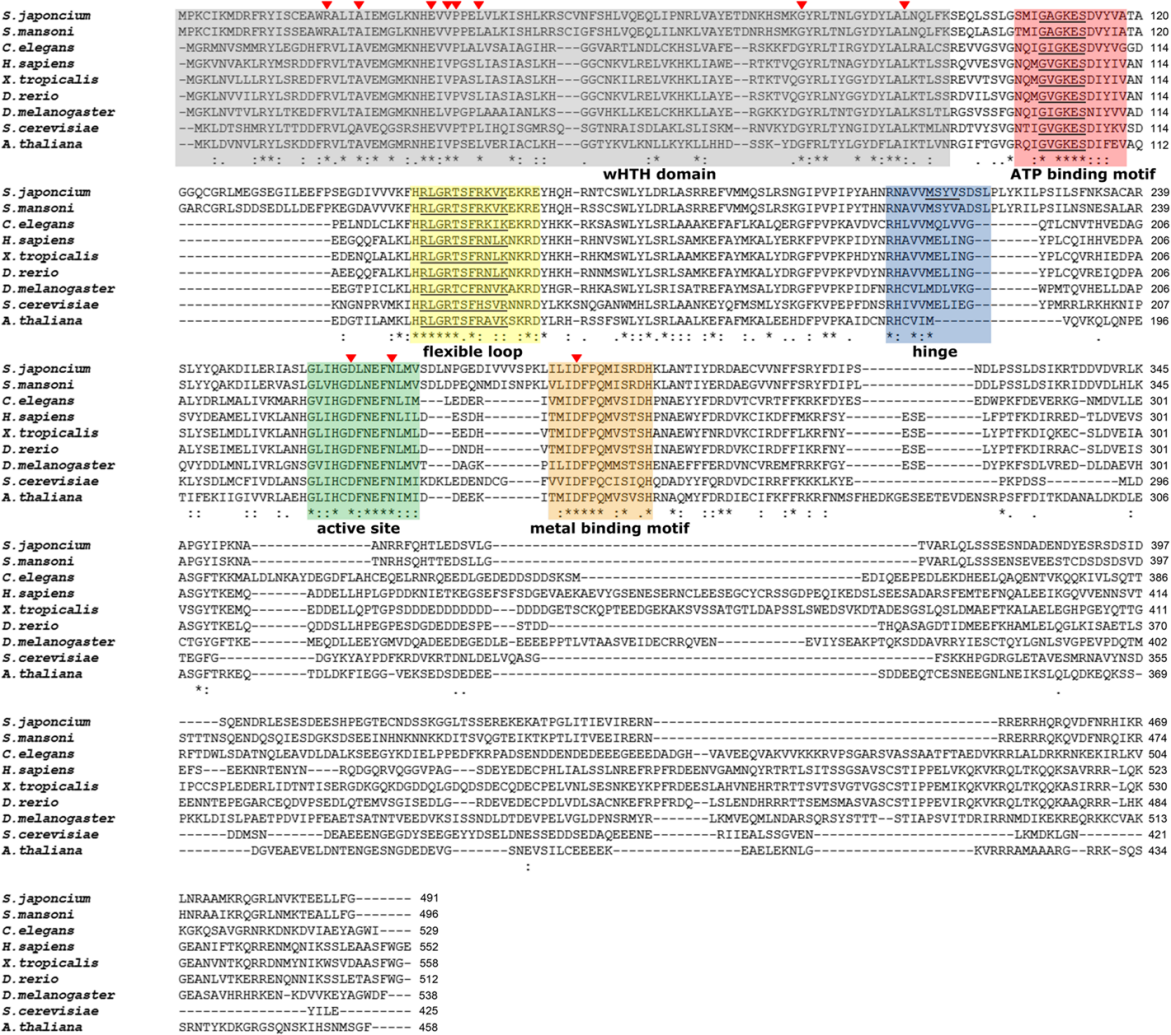
Alignment of the inferred amino acid sequences of *Schistosoma japonicum Sj*-RIOK-2 with RIOK-2 orthologs of eight other species. The eight selected species are *Schistosoma mansoni* (CCD79377.1, *Sm*-RIOK-2), *Caenorhabditis elegans* (CAC70109.2, *Ce*-RIOK-2), *Homo sapiens* (NP_060813.2, *Hs*-RIOK-2), *Xenopus tropicalis* (NP_001016682.1, *Xr-*RIOK-2), *Danio rerio* (NP_998719.2, *Dr.*-RIOK-2), *Drosophila melanogaster* (NP_651365.1, *Dm*-RIOK-2), *Saccharomyces cerevisiae* (CAC70109.2, *Sc*-RIOK-2), *Arabidopsis thaliana* (AEE78772.1, *At*-RIOK-2). Functional domains including the wHTH region (*grey*), ATP binding motif (*red*), flexible loop (*yellow*), hinge region (*blue*), active site (*green*), metal binding motif (*orange*) are highlighted and labeled below the alignment. Red arrows indicate conserved residues within the wHTH domain and “Asp” and “Asn” residues responsible for phosphoryl transfer exits within the active site, and “Asp” required for the positioning of metal ions exits within the metal binding motif. Asterisks under the alignment indicate identical residues. “:” represents strong similarity, “.” represents weak similarity, and a lack of a symbol indicates no similarity among residues

*In silico* analyses showed that the *Sj-*RIOK-2 has high sequence identity to other RIOK-2 orthologs from *S. mansoni* (76%), *S. haematobium* (70%), *C. elegans* (66%), *H. sapiens* (52%) and *D. melanogaster* (50%). Protein sequence alignment (Fig. 2) confirmed that *Sj*-RIOK-2 shares conserved regions, including the N-terminal located winged helix (wHTH) region (amino acid (aa) positions 1–96), an ATP-binding motif (positions 105–118), a flexible loop (positions 151–165), a hinge region (positions 209–221), a catalytically active site (positions 257–270) and a metal binding loop (positions 287–299). In addition, the RIOK-2-specific motif G-x-GKES, which is highly conserved in the N-terminal part of the ATP-binding motif [35], and the autophosphorylation site, which exhibits a slightly altered RLGRTSFRKVK sequence [34, 35], were found in the flexible loop. The key residues “Asp” and “Asn” responsible for phosphoryl transfer, which are involved in catalytic function and are conserved in all ePKs [18, 59], were also found in the active sites of *Sj*-RIOK-2 (D262, N267). A conserved residue “Asp”, required for the positioning of metal ions [35], was detected within metal-binding motif (D290) (Fig. 2).

**Fig. 3:**
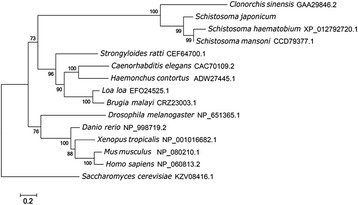
The neighbor-joining (NJ) tree of *Schistosoma japonicum* RIOK-2 with 13 homologues from 13 selected species. The species selected were nine invertebrates including *Brugia malayi* (CRZ23003.1), *Clonorchis sinensis* (GAA29846.2), *Caenorhabditis elegans* (CAC70109.2), *Drosophila melanogaster* (NP_651365.1), *Haemonchus contorts* (ADW27445.1), *Loa loa* (EFO24525.1), *Schistosoma haematobium* (XP_012792720.1), *Schistosoma mansoni* (CCD79377.1), *Strongyloides ratti* (CEF64700.1) and four vertebrates including *Danio rerio* (NP_998719.2), *Homo sapiens* (NP_060813.2), *Mus musculus* (NP_080210.1), *Xenopus tropicalis* (NP_001016682.1). The RIOK-2 from *Saccharomyces cerevisiae* (KZV08416.1) was used as the outgroup. Bootstrap values are displayed above or below the branches

Phylogenetic analyses (Fig. 3) of orthologous amino acid sequences from 13 selected species showed that *Sj*-RIOK-2 grouped together with orthologs from *S. haematobium*, *S. mansoni* and *C. sinensis* with strong (100%) nodal support, to the exclusion of RIOK-2 s from vertebrates, including mammals, fish and amphibians, which also grouped together with high bootstrap support (100%). All RIOK-2 s representing nematodes formed a cluster with 96% support.

### Transcriptional profiling confirmed *Sj-riok-2* transcripts in all life-cycle stages and a gonad-preferential expression in adult females and males

**Fig. 4:**
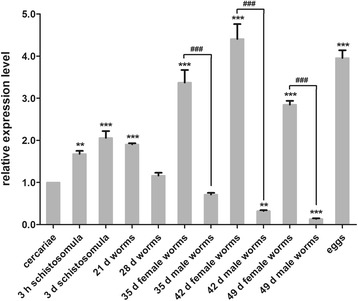
Transcriptional profiles of *Sj-riok-2* detected in different developmental stages of *Schistosoma japoncium* by real-time PCR. Relative expression levels of transcripts were analyzed by the 2^-△△Ct^ method, and *Sj*-*β-tubulin* was used as internal standard. Data are representative of the mean ± SD of three separate experiments. All stages were compared with the cercariae stage, and statistically significant differences are shown as ** (*P* = 0.001) and *** (*P* < 0.0001); ### represents the significant difference (*P* < 0.0001) in transcript abundances between female and male worms

*Sj-riok-2* transcripts were detected in different developmental stages of *S. japonicum* by qRT-PCR, in which cercariae were used as a calibrator (Fig. 4). The results showed that *Sj-riok-2* was most abundantly transcribed in 42 d adult female worms and eggs. The skin- (3 h) and lung- (3 d) stage schistosomula contained a similar abundance of *Sj-riok-2* transcripts as the hepato-portal worms (21 d). Compared with adult males, the transcription level in females was 4.7-, 13.7- and 22-fold higher in 35, 42 and 49 d old worms (*F*_(11,23)_ = 223.75, *P* < 0.0001).

**Fig. 5:**
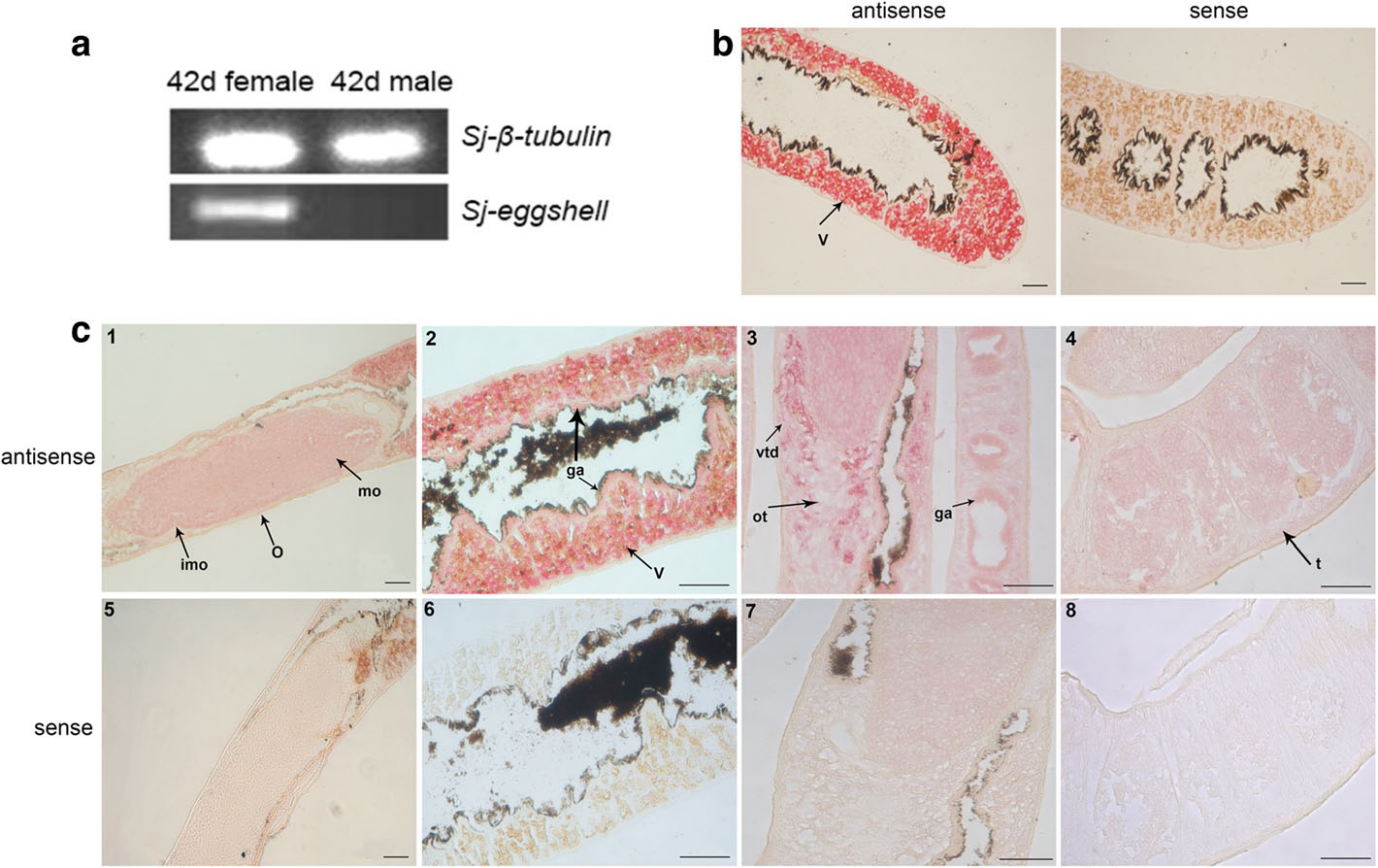
Transcription localization of *Sj-riok-2* detected by *in situ* hybridization on sections of adult worms of *Schistosoma japonicum*. **a** Semi-quantification of the *Sj-eggshell* (*Sj-es*) gene in 42 d adult worms. **b** Histological localization of *Sj-es,* with antisense probe (left) and sense probe (right) of *Sj-es*. **c** Histological localization of *Sj-riok-2*, with antisense probe (c1-4) and sense probe (c5-8). Transcripts of *Sj-es* were detected in the vitellarium (v), and *Sj-riok-2* was localized mainly in all reproductive organs: ovary (o), vitellarium (v), mature oocyte (mo), immature oocyte (imo), vitelloduct (vtd), ootype-surrounding area (ot), testis (t) and gastrodermis (ga). *Scale-bars*: 50 μm

The transcription of *Sj-riok-2* in adult worms was localized by *in situ* hybridization. Employed as a positive control, the eggshell gene of *S. japonicum* (*Sj-es*; p14 ortholog of *S. mansoni*) [57] was only transcribed in mature females (Fig. 5a) and was detected in the vitellarium of adult females (Fig. 5b), which is in accordance with previous reports for *S. japoncium* and *S. mansoni* [57, 60]*. Sj-riok-2* was also detected in the ovary and vitellarium (Figs. 5c-1 and 2). The hybridization signals appeared to be slightly stronger in immature oocytes, which accumulated at the edge of ovary surrounding the large, mature oocytes. In addition, *Sj-riok-2* transcripts were also detected in the gastrodermis and the tissues surrounding the ootype, likely including vitelloduct and oviduct (Figs. 5c-2 and 3). The vitelloduct indicates that mature S4 vitelline cells contain *Sj-riok-2* transcripts. In males, signals were observed in the testes, although signal intensity was weaker compared with tissues in the females (Fig. 5c-4). This result is in accordance to the qPCR data obtained by the stage-specific expression analysis (Fig. 4).

### The transcription level of *Sj-riok-2* is influenced by pairing

During cultivation, the majority of worms remained in a constant pairing contact. Upon perfusion and culturing, single males started to re-pair with the females after 2–4 h, and about 80% worms exhibited a pairing status after 12 h in culture.

**Fig. 6:**
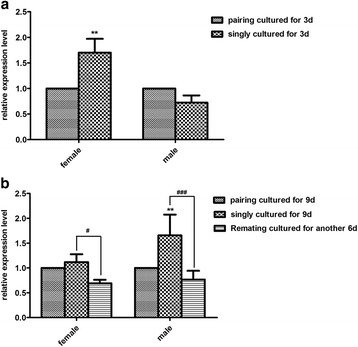
Effects of pairing on the transcript level of *Sj-riok-2* in the female and male *Schistosoma japonicum*. Worm couples, single females, and single males were cultured for 3 d (**a**) and for 9 d (**b**) in vitro. After 3 d, the same number of single females and males were combined for re-pairing for another 6 d (**b**). Singly cultured and re-paired worms were compared with paired worms, and statistically significant differences were shown as ** (*P* < 0.01); #/### represent the significant differences (*P* < 0.05 and *P* < 0.001) in transcript abundances between re-paired worms and single worms

The transcript level of *Sj-riok-2* in single females was 0.8-fold and 0.3-fold higher than in paired females after in vitro culture for 3 d (*F*_(1,7)_ = 7.43, *P* = 0.0295, *t*_(5)_= 6.045, *P *< 0.01) and 9 d (*F*_(2,11) _= 14.02, *P *= 0.0009, *t*_(6)_ = 0.6936, *P >* 0.05), respectively (Fig. 6a). However, the transcription level of *Sj-riok-2* in single males was 0.3-fold lower than in the paired males after culture for 3 d with a non-significant difference (*P* = 0.0759) (Fig. 6a). Nevertheless, the transcription level was 0.7-fold higher in single males after culture for 9 d (*t*_(6)_ = 3.904, *P* < 0.01) (Fig. 6b). Interestingly, the transcript levels of *Sj-riok-2* in single females and males were significantly decreased after re-pairing with the opposite gender for 6 d. It was significantly lower in females and males after re-pairing compared with females (*t*_(6)_ = 2.269, *P* < 0.05) and males (*t*_(6)_ = 5.291, *P* < 0.001) cultured separately (Fig. 6b).

### RNAi-induced knockdown of *Sj-riok-2* demonstrated its involvement in processes controlling mitosis and female gonad-differentiation in adult *S. japonicum*

A gene silencing approach was performed to obtain evidence for the function of *Sj-riok-2*. Due to the presence of transcripts in the gonads, and due to evidence in the literature [42, 43] indicating that RIOK-2 was identified as a substrate of mitotic kinase PLK-1 (Polo-Like Kinase 1) in HeLa cells and can be involved in cell-cycle processes, we considered the possibility that this gene may be involved in reproductive processes such as cell proliferation activities in gonadal cells. In an independent study [24], functional evidence indicated that the activity of the PLK-1-encoding gene *Sm*-*plk-1* is important for cell-cycle progression in gonadal cells of *S. mansoni*. Therefore, we used the PLK-1 encoding gene of *S. japonicum, Sj-plk-1,* as control for comparing effects. The kinase-family member *Sj-stk-6* was used as functionally unrelated negative control.

**Fig. 7:**
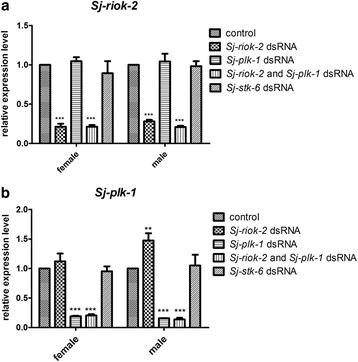
Transcriptional levels of *Sj-riok-2* and *Sj-plk-1* in worms treated with *Sj-riok-2* dsRNA or/and *Sj-plk-1* dsRNA. Relative *Sj-riok-2* (**a**) and *Sj-plk-1* (**b**) transcript levels of females and males, respectively, after treatment with *Sj-riok-2* dsRNA, *Sj-plk-1* dsRNA, or both *Sj-riok-2* and *Sj-plk-1* dsRNAs; as controls *Sj-stk-6* dsRNA or no dsRNA were used. All dsRNA-treated groups were compared with control (non-treated) groups. Data are representative of the mean ± SD of three separate experiments. **, *** indicate *P *< 0.01, *P* < 0.001

Paired worms were treated by soaking with dsRNA targeting *Sj-riok-2* or/and *Sj-plk-1*, *Sj-stk-6* or without dsRNA for 9 d in vitro. Compared with the untreated group (control), the transcript abundances of *Sj-riok-2* in female and male worms treated with dsRNA targeting *Sj-riok-2* alone or with a mixture of dsRNA targeting both *Sj-riok-2* and *Sj-plk-1* decreased by 73–86%, respectively (*F*_(4,20)_ = 83.69, *P* < 0.0001, Fig. 7a). Similarly, transcript abundance of *Sj-plk-1* in female and male worms treated with dsRNA targeting *Sj-plk-1* alone or with a mixture of dsRNA targeting both *Sj-plk1* and *Sj-riok-2* decreased by 80–91%, respectively (*F*_(4,20)_ =72.73, *P* < 0.0001, Fig. 7b). However, although treatment with dsRNA targeting *Sj-plk-1* had no effect on transcription levels of *Sj-riok-2* in worms, treatment with dsRNA targeting *Sj-riok-2* alone caused an increase of 50% of the transcript levels of *Sj-plk-1* in male worms (*t*_(6)_ = 3.864, *P* < 0.01). No significant differences were detected between the control and the non-specific (*Sj-stk-6*) dsRNA groups.

**Fig. 8:**
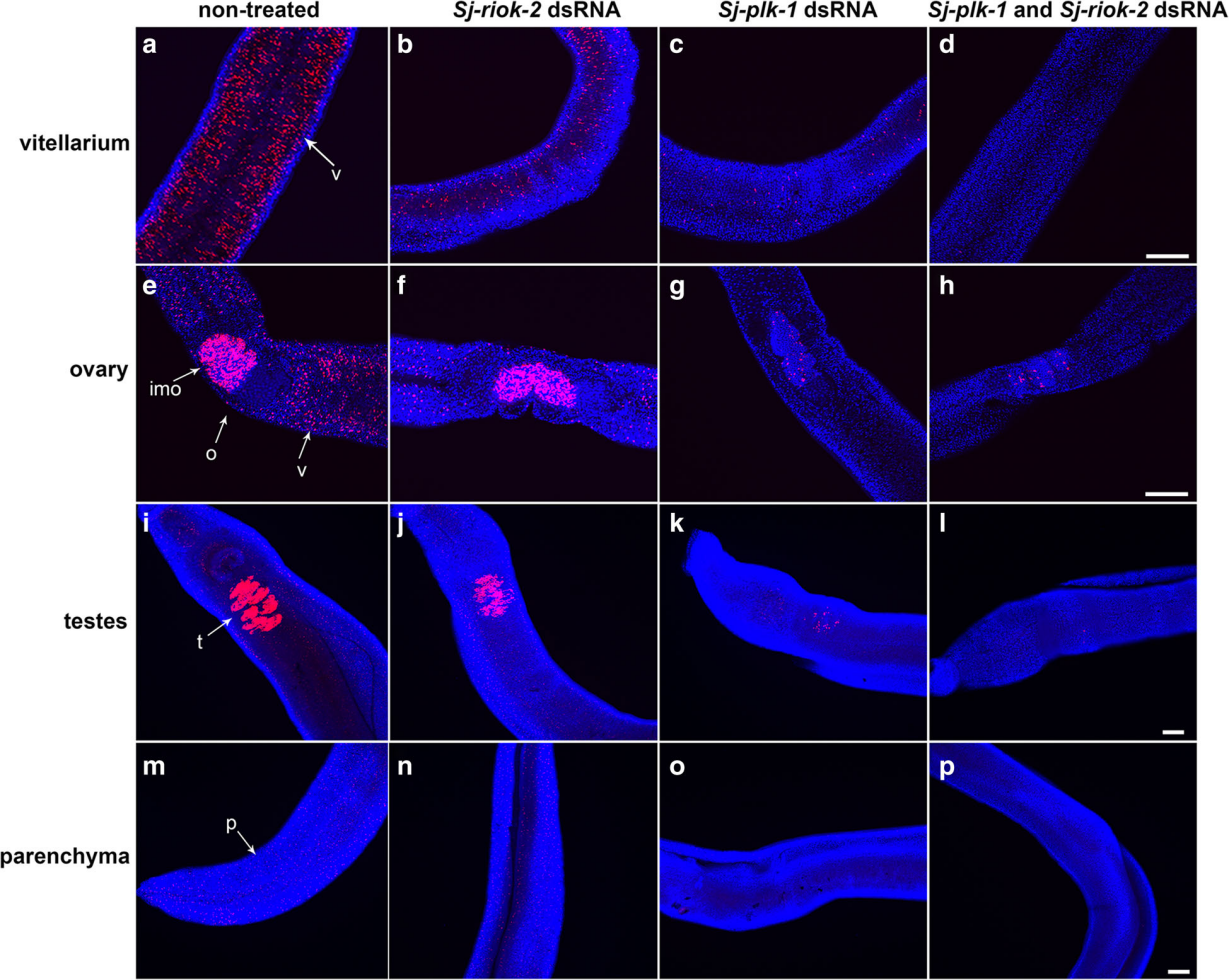
Cell proliferation activity of *Schistosoma japonicium* couples treated with dsRNAs of *Sj-riok-2* and/or *Sj-plk-1.* The EdU-incorporation cells of non-treated worms were detected in the vitellarium and ovary of females (**a**, **e**), testes and parenchyma of males (**i**, **m**). EdU+ cells were detected in *Sj-riok-2* dsRNA (**b**, **f**, **j**, **n**)-, *Sj-plk-1 *dsRNA (**c**, **g**, **k**, **o**)-, *Sj-riok-2-* and *Sj-plk-1* dsRNA-group (**d**, **h**, **l**, **p**). *Abbreviations*: v, vitellarium; o, ovary; imo, immature oocytes; t, testes; p, parenchyma. *Scale-bars*: 50 μm

To investigate whether *Sj-riok-2* is involved in processes controlling mitoses, EdU-labeling was performed with dsRNA-treated worms as a technique to follow cell proliferation [58]. In an untreated control group, a significant amount of EdU-labeled cells were detected in the vitellarium and ovary of adult females as well as in the testis and parenchyma of adult males (Fig. 8a, e, i, m), which indicated high mitotic activity in these organs. Adult worms treated with *Sj-riok-2* dsRNA for 9 d showed a slight decrease of EdU-positive cells in the vitellarium of the female and the testis and parenchyma of the male (Fig. 8b, f, j, n). However, the number of EdU-labeled cells was remarkably reduced in the vitellarium, ovary, testis and parenchyma of worms treated with *Sj-plk-1* dsRNA for 9 d (Fig. 8c, g, k, o). Worms treated with both *Sj-riok-2* and *Sj-plk-1* dsRNA had almost no EdU-labeled cells in these organs and tissue (Fig. 8d, h, l, p). A previous study has shown that developmental failures in vitellarium were due to an increase of cell apoptosis rather than decreased cell proliferation [61]. Therefore, the transcription levels of three apoptosis-associated genes of *S. japonicum*, caspase 3, caspase 7 and cytokine apoptosis inhibitor (CIAP) [62], were quantified after knocking down *Sj-riok-2* or/and *Sj-plk-1*. The results showed that knocking down *Sj-riok-2* had no significant impact on the transcriptional levels of the genes *Sj-caspase-3* (*F*_(3,8__)_= 0.089, *P *= 0.964), *Sj-caspase-7* (*F*_(3,8__)_= 0.457, *P *= 0.720) and *Sj-ciap* (*F*_(3,8)_ = 0.524, *P* = 0.678*)* (Additional file 2: Figure S1).

**Fig. 9:**
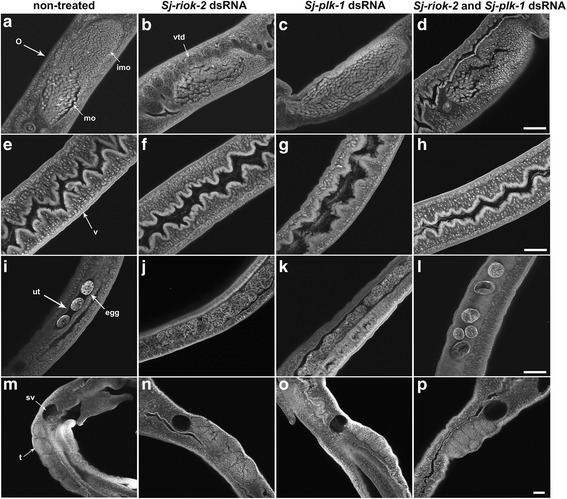
Morphology of the reproductive organs from *Schistosoma japonicium* couples treated with dsRNAs of *Sj-riok-2* and/or *Sj-plk-1.* Whole mount preparations of non-treated worms (**a**, **e**, **i**, **m**), *Sj-riok-2* dsRNA-treated worms (**b**, **f**, **j**, **n**), *Sj-plk-1* dsRNA- treated worms (**c**, **g**, **k**, **o**) and *Sj-riok-2* plus *Sj-plk-1* dsRNA doubly-treated worms (**d**, **h**, **l**, **p**) were observed by confocal scanning laser microscope. *Abbreviations*: o, ovary; imo, immature oocytes; mo, mature oocytes; v, vitellarium; vtd, vitelloduct; ut, uterus; t, testes; sv, sperm vesicle. *Scale-bars*: 100 μm

Morphological analysis by CLSM finally showed no detectable changes in the size of adult worms, and the pairing stability of couples was unaffected for worms treated with *Sj-riok-2* and *Sj-plk-1* dsRNA alone or simultaneously (Additional file 3: Figure S2). However, morphological changes were observed in the gonads of adult females treated with dsRNA for 9 d (Fig. 9). Although in untreated control females, small immature oocytes occur in the anterior part and larger mature oocytes in the posterior part of the ovary (Fig. 9a), the number of mature oocytes increased and dominated most of the ovary in *Sj-riok-2* dsRNA-treated females (Fig. 9b). A similar trend was seen in the females treated with *Sj-plk-1* dsRNA, where the number of immature oocytes dramatically declined while the mature oocytes were distributed throughout the ovary (Fig. 9c). Unexpectedly, females treated with both *Sj-riok-2* and *Sj-plk-1* dsRNA showed a similar proportional distribution of oocytes in their ovaries as control worms, with the exception of the smaller size of the ovary (Fig. 9d). Furthermore, females treated with *Sj-riok-2* dsRNA showed a dilated vitelloduct, which undulates alongside the ovary (Fig. 9b). Within the vitellarium of females treated with *Sj-plk-1* dsRNA, the number of vitelline cells decreased, and these cells appeared to be irregular and disordered (Fig. 9g). However, no significant morphological changes were observed in the females treated with both dsRNAs compared to the controls (Fig. 9h). Moreover, compared to control females (Fig. 9i), more eggs were observed inside the uterus of worms treated with *Sj-riok-2* dsRNA or *Sj-plk-1* dsRNA alone (Fig. 9j, k). Interestingly, although the amount of eggs in the uterus of the females treated with both dsRNAs was similar to the control, more abnormal eggs were found in these females (Fig. 9l). No visible content was observed in such eggs, and they seemed to be non-embryonated. However, no significant difference was found in the number of eggs laid in vitro by untreated and dsRNA-treated females (Additional file 4: Figure S3). In addition, no visible phenotype was observed in the testes or the sperm vesicle of male worms treated with any dsRNA (Fig. 9m-p).

## Discussion

In the present study, the RIOK-2 encoding gene *Sj-riok-2* of *S. japonicum* was cloned and characterized. Alignment of the protein sequence of *Sj*-RIOK-2 with orthologous sequences of other species revealed that typical functional domains of this class of enzymes are also present in *Sj*-RIOK-2. Unlike ePKs, RIOK-2 lacks the activation loop that functions in substrate binding and recognition [63]. However, the potential substrate-binding regions predicted for the catalytic domain of *Plasmodium* RIOK-2 [64] are present in *Sj*-RIOK-2 such as the “H-R-L-G-R-I/T” motif within the flexible loop region and the M-S-Y-I/V motif within the hinge region. *Sj*-RIOK-2 exhibits a wHTH domain, which usually occurs in transcription factors or other DNA-binding proteins [65, 66]. It has been shown previously that the wHTH domain of *Archaeoglobus fulgidus* RIOK-2 can nonspecifically bind to single-strand oligonucleotides [63]. In addition, as the wHTH domain of *Plasmodium* RIOK-2 can interact with its kinase domain, it has been proposed that the interaction between DNA/RNA and wHTH could function in ribosome maturation by affecting RIOK-2 kinase activity [64]. The conservation of these regions in *Sj*-RIOK-2 suggests that *Sj*-RIOK-2 may have similar functions in *S. japonicum*.

In parasitic nematodes, the transcriptional profiles of *riok-2* at different developmental stages have been analyzed for several species including *H. contortus*, *B. malayi* and *Ascaris suum* [44, 67]. In addition, in the free-living nematode *C. elegans*, silencing *riok-2* resulted in embryonic lethality and early larval arrest [46, 47], suggesting the importance of RIOK-2 for larval growth and development. Moreover, in *Drosophila*, the elevated transcript levels of *riok-2* in ovary likely contribute to the higher transcriptional level of *riok-2* in females than males [68], indicating that *riok-2* might be involved in the reproductive processes [67]. In the present results, transcriptional profiling indicated that *Sj-riok-2* is transcribed in all developmental stages of *S. japonicum*. Compared with male worms, higher transcription levels were detected in females and eggs. Furthermore, in adult females, *Sj-riok-2* was mainly localized in the reproductive organs, suggesting that *Sj-riok-2* could be involved in female reproductive biology in *S. japonicum*. Previous studies [69–71] have indicated an influence of pairing on the expression of genes in schistosome females, particularly genes with functions in the reproductive organs. Pairing experiments in our study showed that, compared with paired females, the transcription level of *Sj-riok-2* was higher in unpaired females than in re-paired females. Thus, it seems likely that *Sj-riok-2* is involved in pairing-influenced processes associated with female maturation and sexual maintenance.

There are conflicting results from the literature about the role of RIOK-2 kinases with respect to mitogenic activity. The depletion of RIOK-2 in yeast did not trigger cell-cycle arrest in any stage [38]. In HeLa cells, knockdown of *riok-2* was found to accelerate mitotic exit, whereas its over-expression did not affect mitotic entry [42]. In a *Drosophila* glioblastoma model, suppression of *riok-2* caused mitotic entry arrest and apoptosis by inducing p53 activity [43]. In first experiments using the *Xenopus* oocyte system, which had been shown before to be suitable for studying kinases [16, 21, 22], it was shown that *Sj-riok-2* delayed the G2-M transition during meiosis using germinal vesicle breakdown assays as read out (Additional file 5: Figure S4). So knockdown *Sj-riok-2* may resume or accelerate meiosis/mitosis entrance. The observation that the number of EdU-labeled cells is less abundant in the vitellarium and testes following knockdown of *Sj-riok-2* suggests that this kinase can influence the cell cycle at the G1 and/or S level. Therefore, we hypothesize that *Sj-riok-2* acts in dividing cells as a balancing element to avoid premature mitosis. Also in the ovary a slight decrease in EdU-positive cells was observed which, however, was not as high as in the other gonad tissues. This indicates that *Sj-riok-2* may fulfill a function in all schistosome tissues with high proliferative capacity. However, this effect could not be completely monitored by EdU labeling that concerns only the S phase. In contrast, the *plk-1* knockdown completely inhibits EdU labeling because PLK-1 strongly controls S phase.

The hypothesis of *Sj-riok-2* as a balancer of mitosis is supported by studies in HeLa cells, in which *riok-2* knockdown accelerated mitotic exit [42]. This indicated that the loss of *riok-2* function accelerated cell-cycle progression to promote post-mitotic differentiation events. In *S. japonicum*, after meiosis, small oogonia develop to primary mature oocytes with a central nucleus that dominate in the posterior part of the ovary. Upon *Sj-riok-2* knockdown, the number of mature oocytes increased. This suggests that less oogonia entered mitotic division ahead of meiosis I and that knockdown of *Sj-riok-2* could promote oocyte maturation towards primary oocytes. In addition, the dilated vitelloduct found upon knockdown may result from an increased number of S4-stage mature vitellocytes, which provide material and nutrition for more eggs production. Compared with the control group, more eggs accumulated in uterus of *Sj-riok-2* dsRNA-treated females. However, there was no significant difference in the number of eggs laid in vitro between the control group and the *Sj-riok-2* dsRNA-treated group. This can be explained by an increased capacity of egg formation, without ovulation being able to keep pace with egg production, leading to egg accumulation in the uterus. Finally, *Sj-riok-2* has also been localized to the ootype-surrounding area, which suggests an additional role of this kinase for egg-formation processes*.*

A previous study proposed that, as an important regulator of Akt signaling pathway, RIOK-2 might act as a ribosomal stress checkpoint to either progress the cell cycle after the completion of ribosomal maturation or activate the apoptosis pathway upon disruption of ribosomal biosynthesis [43, 67]. However, knocking down *Sj-riok-2* had no significant impact on the transcriptional levels of three apoptosis-associated genes in *S. japonicum*, suggesting that *riok-2* could be involved in oocytes or vitelline cell maturation that was independent of activating apoptosis. In the cell cycle, the activation of anaphase-promoting complex or cyclosome (APC/C), a conserved multi-subunit E3 ubiquitin (Ub) ligase is required for promoting anaphase initiation and mitosis exit, but preventing meiotic resumption in prophase I-arrested mouse oocytes by degrading its numerous substrates [72–77]. RIOK-2 was identified to interact with APC complex subunits, Apc10 and Apc3 [42], and Apc10 interacting with Apc3 [78]. In addition, Apc10 and Apc3, as important substrate-and activator-binding subunits, respectively [79, 80], assembled with catalytic core member Apc 11 and then contributed to the substrate ubiquitination [74, 81, 82], suggesting that RIOK-2 might be a part of APC/C complex core targeting substrates or proteins [42]. According to the above results, we assume that *Sj*-RIOK-2 may activate APC/C complex to degrade cyclin B, then induced G2-M transition arrested. This was also in consistent with the result that *Sj-riok-2* delayed meiotic resumption in *Xenopus* oocytes.

Previous studies indicated roles of PLK-1 in multiple mitotic events such as CDK1 activation, centrosome maturation, spindle assembly and APC/C regulation [83]. Inhibition of *plk-1* by specific inhibitors or RNAi arrested G2/M transition in human cells [84] and *Xenopus* oocytes [24], indicating that the suppression of *plk-1* induced the termination of cell proliferation by arresting both mitotic and meiotic progresses. In HeLa cells, PLK-1 can phosphorylate RIOK-2 to regulate mitotic progression [42]. In *S. mansoni*, *Sm*-PLK-1 was identified as a mitotically active kinase, it was mainly localized in the reproductive organs and shown to be required for oogenesis and spermatogenesis [24]. With respect of the co-localization in the gonads, a cooperation of both molecules seemed possible. Knocking down *Sj-plk-1* induced a significant reduction of EdU-labelled cells in the gonads, because cell proliferation was inhibited. A similar, but much weaker effect was observed upon silencing *Sj-riok-2* activity. In *Sj-riok-2* and *Sj-plk-1* double knockdown worms, the smallest number of EdU-labeled cells was detected. These findings point to an additive effect, indicating that both molecules appear to cooperate. Previously it was found that PLK-1 could mediate the phosphorylation of RIOK-2 to regulate mitotic exit [42]. This observation supports the proposal of a cooperating role of both proteins, whereas PLK-1 might have a superior function in supporting mitotic processes.

Finally, CLSM analyses demonstrated that in *Sj-plk-1* dsRNA-treated females, the number of mature oocytes had increased compared to controls. This phenotype is consistent with the morphological changes observed inside the ovary of *S. mansoni* females treated with a PLK-1 inhibitor, which probably caused a mitotic arrest and the termination of oogonia divisions [24, 84]. Given that knockdown of *Sj-plk-1* did not affect the transcription level of apoptosis-associated genes (Additional file 2: Figure S1), the significant reduction of the number of oogonia might have been caused by an arrested cell cycle, while post-mitotic, differentiating oocytes expand into the complete cavity of the ovary. Although a similar phenotype was also observed in *S. japonicum* females after knocking-down *Sj-riok-2*, it was not as strong as that seen for PLK-1*.*Thus we assume that *Sj-riok-2* and *Sj-plk-1* may play cooperating roles in cell-cycle control in the gonads, and *Sj-riok-2* may counter balance the activity of *Sj-plk-1.* The similar gonad morphologies of females of the double knockdown and control groups support this hypothesis.

## Conclusions

This study represents the first functional characterization of RIOK-2 in platyhelminthes. We cloned and characterized a RIO-2 kinase encoding gene from *S. japonicum* (*Sj-riok-2*). It exhibits typical structural features of orthologs from other eukaryotic species and *Sj-riok-2* is transcribed in different developmental stages of *S. japonicum* with highest abundance in 42 day-old females. In both males and females, transcription occurred pairing-dependently, and transcripts were localized mainly to the reproductive organs. Knocking-down *Sj-riok-2* reduced cell proliferation in the gonads and caused an increase of the amount of mature oocytes in the ovary as well as an accumulation of eggs in the uterus. Thus, our data demonstrate that *Sj-riok-2* is involved in key processes controlling the reproductive biology of female *S. japonicum.*

## Additional files

Additional file 1: Table S1. DNA sequences of oligonucleotide primers used in the present study. These primers were employed for the isolation of 3'cDNA of *Sj-riok-2* and for the amplification of DNA template for dsRNA synthesis of *Sj-riok-*2, *Sj-plk-1* and *Sj-stk-6* genes using PCR-based approaches and for real-time (RT) PCR quantification. (DOCX 14 kb)

Additional file 2: Figure S1. Transcriptional levels of the apoptosis-associated genes caspase 3, caspase 9 and cytokine apoptosis inhibitor (CIAP) in female *S. japonicum* treated with specific *Sj-riok-2* dsRNA or/and *Sj-plk-1* dsRNA. Relative *Sj-caspase-3, Sj-caspase-9* and *Sj-ciap* transcript levels of females after treatment with *Sj-riok-2* dsRNA, *Sj-plk-1* dsRNA or both *Sj-riok-2* and *Sj-plk-1* dsRNA were detected by RT-PCR, respectively. Data are representative of the mean ± SD of three separate experiments. (TIFF 167 kb)

Additional file 3: Figure S2. Comparison of the worm length between the control group and *Sj-riok-2* dsRNA- or/and *Sj-plk-1* dsRNA-treated group. The body length of female and male worms was measured under microscope after in vitro culture for 9 d, respectively. No significant difference was found among the different groups. Data are representative of the mean ± SD of three separate experiments. (TIFF 607 kb)

Additional file 4: Figure S3. Egg count upon RNAi treatment. The numbers of eggs laid ex vivo by female worms from the control group and the *Sj-riok-2* dsRNA- or/and *Sj-plk-1* dsRNA-treated groups during 3–5 days, 5–7 days, 7–9 days were counted manually. No significant differences were detected among these groups. Data are representative of the mean ± SD of three separate experiments. (TIFF 469 kb)

Additional file 5: Figure S4. *Sj-riok-2* activity in *Xenopus* oocyte germinal vesicle break down (GVBD) assays. Capped messenger RNA (cRNA) of *Sj-riok-2* was microinjected into *Xenopus laevis* stage VI oocytes according to a standard protocol [21, 22]. Each oocyte was injected with 60 nl (60 ng) cRNA in the equatorial region and incubated at 19 °C in ND96 medium. Results are expressed as the percentages of the number of mature oocytes found in samples injected with *Sj-riok-2* cRNA or uninjected. After 10 h, GVBD was detected in all uninjected oocytes by the appearance of a white spot at the center of the animal pole. Compared to the uninjected group, the GVBD oocytes were decreased in *Sj-riok-2* cRNA injected oocytes. (TIFF 35 kb)

## Abbreviations

aPK: Atypical protein kinase
CLSM: Confocal laser scanning microcopy
EdU: 5-ethynyl-2′-deoxyuridine
ePK: Eukaryotic protein kinase
RIO: Right open reading frame
