# Supplementary material for: The RIO protein kinase-encoding gene Sj-riok-2 is involved in key reproductive processes in Schistosoma japonicum
Source: Parasit Vectors. 2017 Dec 12;10:604. doi: 10.1186/s13071-017-2524-7 (PMC5727939; doi:10.1186/s13071-017-2524-7)
Supplement: Supplementary file 1 — DNA sequences of oligonucleotide primers used in the present study. These primers were employed for the isolation of 3'cDNA of Sj-riok-2 and for the amplification of DNA template for dsRNA synthesis of Sj-riok-2, Sj-plk-1 and Sj-stk-6 genes using PCR-based approaches and for real-time (RT) PCR quantification. (DOCX 14 kb) [file 13071_2017_2524_MOESM1_ESM.docx]

**Additional file 1: Table S1**. DNA sequences of oligonucleotide primers used in the present study. These primers were employed for the isolation of 3’cDNA of *Sj-riok-2* and for the amplification of DNA template for dsRNA synthesis of *Sj-riok-*2, *Sj-plk-1,* and *Sj-stk-6* genes using PCR-based approaches and for real-time (RT) PCR quantification.

| Primer name | Sequence (5’-3’) |
| --- | --- |
| Sjriok2-1F | AGTCGTTCCTCCTGAGCTGGTGC |
| Sjriok2-3F | GGCTATCGTCTTACCAACTTGGG |
| Sjriok2-ORF-F | TCGAGGATCCATGCCAAAGTGTATAAAAATGGATCGC |
| Sjriok2-ORF-R | CACGGTCGACTTATCCGAAAAGCAATTCTTCAGTTT |
| Riok2-F | TGGACAATGTGGACGACTAAT |
| Riok2-R | GCTGGTGATACTCCCTTTTTT |
| Plk1-F | TAAGCCTAAAGATCCACCGG |
| Plk1-R | ACCAGCTCGTAGCATTTTGC |
| β-Tubulin-F | GCGGGACAGTGTGGTAATCA |
| β-Tubulin-R | ATGCGTTCAAGTTGTAAATCAGAG |
| ISH-es-F | ATTTAGGTGACACTATAGAACCTACCCACCATCATCCGA |
| ISH-es-R | TAATACGACTCACTATAGGGTTTCCGTAGCTGTCGCCAC |
| ISH-riok2-F | ATTTAGGTGACACTATAGAACAGTTATCGTCATCAGAAAG |
| ISH-riok2-R | TAATACGACTCACTATAGGGTTCACATTAAGACGACCCT |
| dsRNA-riok2-F | TAATACGACTCACTATAGGGCAGTGTATTTATTCTTCGGC |
| dsRNA-riok2-R | ATTTAGGTGACACTATAGAAAAGGTAAGCAGTTAGGCAG |
| dsRNA-plk1-F | TAATACGACTCACTATAGGGTGTAGGAGCTATCTTCGTGTTTGG |
| dsRNA-plk1-R | ATTTTAGGTGACACTATAGAAGAGGATTCGCAAAATGCTACGAGC |
| dsRNA-stk6-F | TAATACGACTCACTATAGGGAGTTTGGATGCTTGGTATG |
| dsRNA-stk6-R | ATTTAGGTGACACTATAGAAAAGAACGAAATGTAGGACG |
