# Supplementary figures and images for: The RIO protein kinase-encoding gene Sj-riok-2 is involved in key reproductive processes in Schistosoma japonicum
Source: Parasit Vectors. 2017 Dec 12;10:604. doi: 10.1186/s13071-017-2524-7 (PMC5727939; doi:10.1186/s13071-017-2524-7)

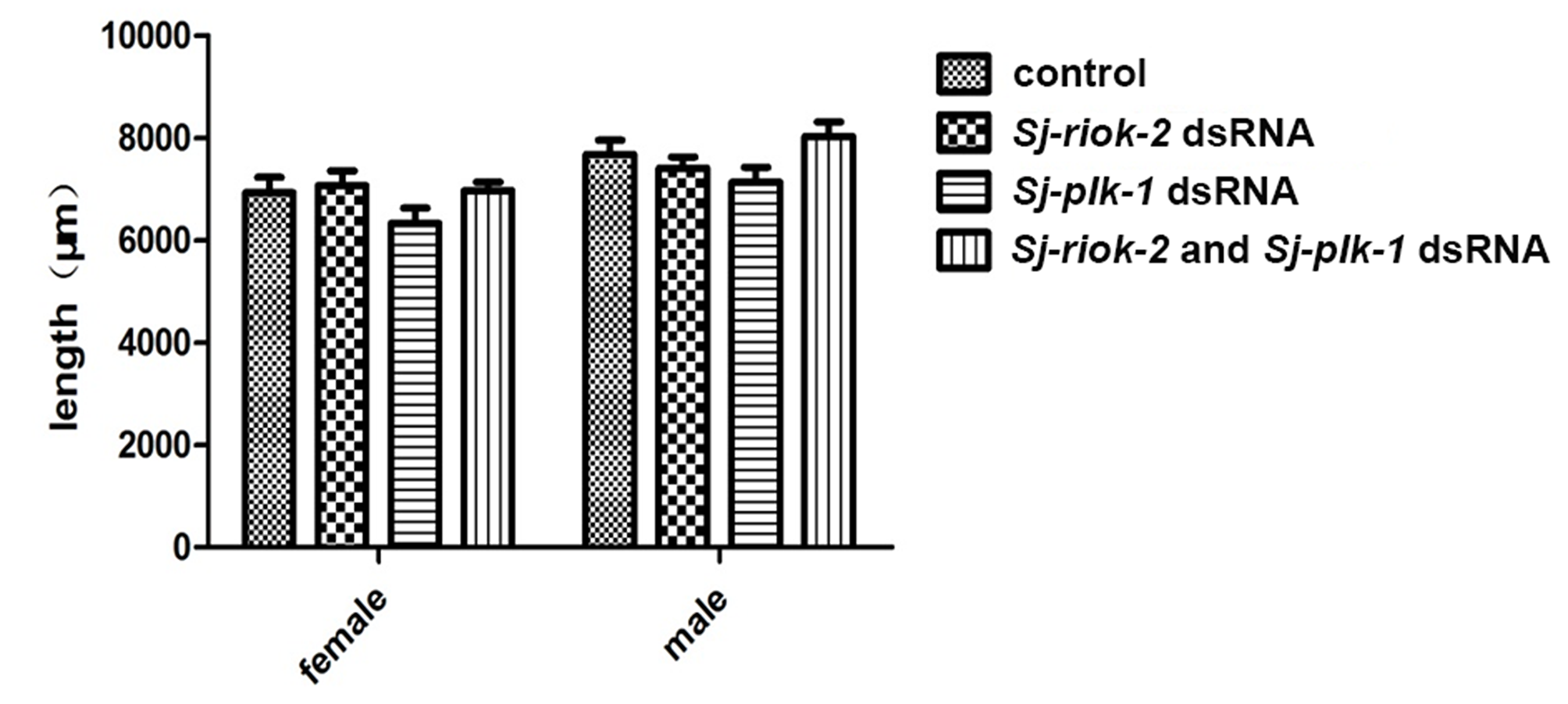

Supplement: Supplementary file 3 — Comparison of the worm length between the control group and Sj-riok-2 dsRNA- or/and Sj-plk-1 dsRNA-treated group. The body length of female and male worms was measured under microscope after in vitro culture for 9 d, respectively. No significant difference was found among the different groups. Data are representative of the mean ± SD of three separate experiments. (TIFF 607 kb) [file 13071_2017_2524_MOESM3_ESM.tif]

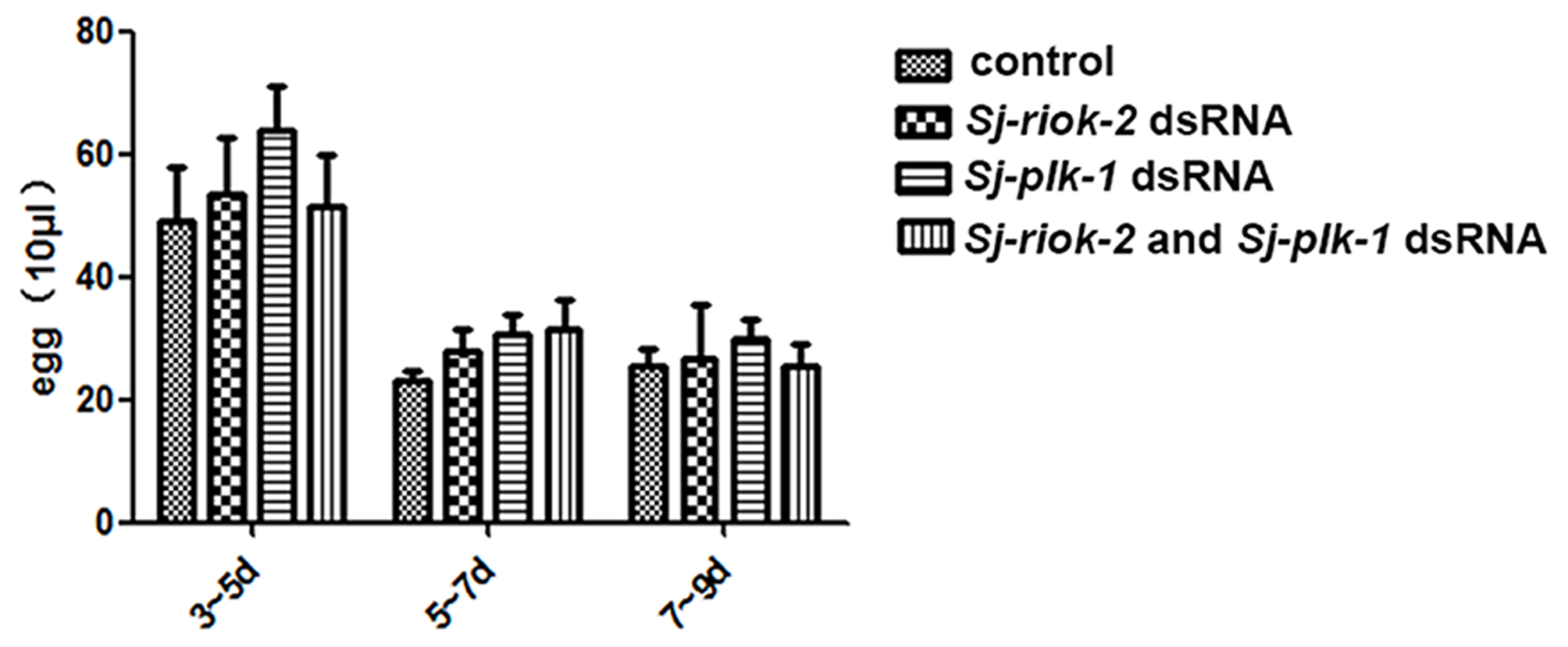

Supplement: Supplementary file 4 — Egg count upon RNAi treatment. The numbers of eggs laid ex vivo by female worms from the control group and the Sj-riok-2 dsRNA- or/and Sj-plk-1 dsRNA-treated groups during 3–5 days, 5–7 days, 7–9 days were counted manually. No significant differences were detected among these groups. Data are representative of the mean ± SD of three separate experiments. (TIFF 469 kb) [file 13071_2017_2524_MOESM4_ESM.tif]

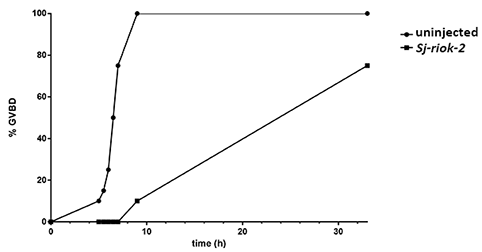

Supplement: Supplementary file 5 — Sj-riok-2 activity in Xenopus oocyte germinal vesicle break down (GVBD) assays. Capped messenger RNA (cRNA) of Sj-riok-2 was microinjected into Xenopus laevis stage VI oocytes according to a standard protocol [21, 22]. Each oocyte was injected with 60 nl (60 ng) cRNA in the equatorial region and incubated at 19 °C in ND96 medium. Results are expressed as the percentages of the number of mature oocytes found in samples injected with Sj-riok-2 cRNA or uninjected. After 10 h, GVBD was detected in all uninjected oocytes by the appearance of a white spot at the center of the animal pole. Compared to the uninjected group, the GVBD oocytes were decreased in Sj-riok-2 cRNA injected oocytes. (TIFF 35 kb) [file 13071_2017_2524_MOESM5_ESM.tif]
